# Precaution against Cholera

**Published:** 1885-02

**Authors:** 


### PRECAUTIONS AGAINST CHOLERA.

In lecturing on the history of international law and custom on the subject, Mr. Hart analyzed the results of the Vienna convention, and discussed separately the practices of European and Transatlantic nations in dealing with cholera. He urged that the evidence was overwhelming that European quarantine by sea, and land quarantine in any case, had invariably proved not only useless in preventing the extension of disease and loss of life, but cruel and mischievous, and had greatly added to the misery and suffering due to outbreaks of cholera. He condemned the attempts at quarantine practiced in France, Italy and Spain, as being contrary to the experience and the knowledge of facts, as well as of science. Quarantine, he maintained, had never kept cholera out of any European country, or limited it in any European district.

He proceeded to describe in detail the system of medical inspection at ports and termini, by which alone, he said, reasonable efforts might be made to prevent or limit the importation of cholera. Governments had practiced innumerable follies and insanities of quarantine, totally contrary to the rules of science, during the last epidemic. Rome, with its pure supply of water and its relatively efficient drainage, had remained free from cholera, while Naples, with its ground soil impregnated with sewage and its filthy habitations and polluted water supply, had suffered most lamentable losses. He had most excellent reasons for believing that the recent outbreak in Paris was due to the temporary supply of a highly polluted water to particular districts of the city. The prevalence of typhoid was, he declared, the true index of the liability to Asiatic cholera. Wherever typhoid prevailed, there the local conditions existed which would favor the propagation of cholera; and until typhoid fever disappeared from among us we could not consider ourselves free from the risk of the importation and the propagation of this epidemic disease. The lessons he desired to urge were :

1. That quarantine was useless. 2. That medical inspection of ports was essential, and with this should go means of isolation, compulsory notification of infectious disease, and the active exertion of all local authorities to free the districts under their control from the known conditions which render them liable to the extension of epidemic diseases when imported. 3. That disinfection was of most doubtful value under the known conditions of such disease. 4. That cleanliness in its fullest and widest sense was the prime element of safety.—*Scientific American.*

